# The First Cytogenetic Data on *Strumigenys louisianae* Roger, 1863 (Formicidae: Myrmicinae: Dacetini): The Lowest Chromosome Number in the Hymenoptera of the Neotropical Region

**DOI:** 10.1371/journal.pone.0111706

**Published:** 2014-11-07

**Authors:** Ana Paula Alves-Silva, Luísa Antônia Campos Barros, Júlio Cézar Mário Chaul, Silvia das Graças Pompolo

**Affiliations:** 1 Laboratório de Citogenética de Insetos, Departamento de Biologia Geral. Universidade Federal de Viçosa; Viçosa, Minas Gerais, Brazil; 2 Programa de Pós-graduação em Genética e Melhoramento. Universidade Federal de Viçosa; Viçosa, Minas Gerais, Brazil; 3 Departamento de Biologia Geral. Universidade Federal de Viçosa; Viçosa, Minas Gerais, Brazil; Queensland Institute of Medical Research, Australia

## Abstract

In the present study, the first cytogenetic data was obtained for the ant species *Strumigenys louisianae*, from a genus possessing no previous cytogenetic data for the Neotropical region. The chromosome number observed was 2n = 4, all possessing metacentric morphology. Blocks rich in GC base pairs were observed in the interstitial region of the short arm of the largest chromosome pair, which may indicate that this region corresponds to the NORs. The referred species presented the lowest chromosome number observed for the subfamily Myrmicinae and for the Hymenoptera found in the Neotropical region. Observation of a low chromosome number karyotype has been described in *Myrmecia croslandi*, in which the occurrence of tandem fusions accounts for the most probable rearrangement for its formation. The accumulation of cytogenetic data may carry crucial information to ensure deeper understanding of the systematics of the tribe Dacetini.

## Introduction

The order Hymenoptera is one of the most diversified among insects and has interesting characteristics regarding its form of reproduction, sex determination and evolution of social behavior [1].

The Minimum Interaction Theory formulated by Imai et al. [2], is well accepted as an explanation for the chromosomal evolution of Formicidae. This theory is based on the occurrence of chromosomal rearrangements, where centric fission is the most frequent event. It postulates that there is a tendency for reduced chromosome size, and consequently an increase in chromosome number by means of fissions and a subsequent heterochromatin growth [2]–[4]. This process is evolutionarily favored by decreasing the interaction between the chromosomes,in particular, the deleterious translocations during meiosis. However, different chromosomal rearrangements have already been reported in ants, including centric fusions (reviewed in [4]) [5], [6].

A wide variation in the chromosome number is observed in Hymenoptera, particularly in the family Formicidae, which includesextremes of variation in the order [4]. This variation ranges from n = 1 in *Myrmecia croslandi*, Australia [7], to n = 60 in *Dinoponera lucida*, Brazil [8]. Among the 750 ant species that have had their karyotypes described, 72 belong to the Neotropical region [4]. This region hosts approximately 3,100 described species and is considered one of the richest in ant species in the world [9].

Currently, the various synonyms related to the genus *Strumigenys* proposed by Baroni Urbani & De Andrade [10] are well accepted [11], although, the proposal of merging the tribes Phalacromyrmecini and Basicerotini into the tribe Dacetini is still controversial [12]. Commenting on this issue is not withinthe scope of this paper and assuming that the Basicerotini and Phalacromyrmecini continue to be ranked as separate tribes [12], the tribe Dacetini would include 203 Neotropical species with representatives from three genera: *Acanthognathus*, *Daceton* and *Strumigenys* [9], [13]–[17]. The genus *Strumigenys* includes 194 species in the Neotropics, although none have been subjected to cytogenetic studies [4], [9]. However, information regarding their chromosome number is available for a few species of this genus from southern Asia and Oceania: *S. doriae* (2n = 22), *S. friedae* (2n = 24) and *S. godeffroyi* (2n = 40, 44) [18]–[20], *Strumigenys* spp. (2n = 16; 2n = 38; n = 13), *S. mutica* (2n = 36) and *S. dohertyi* (2n = 24) (in [4], as *Pyramica* spp., *P. mutica* and *P. dohertyi*, respectively).

*Strumigenys louisianae* distribution ranges from southern United States to Argentina. This species shows great morphological variation, including the density and intensity of the sculpture on the mesosoma, on the post-petiole and gaster and the shape and size of the spongiform appendages, possiblybeing that *S. louisianae* represents, in fact, a complex of species according to the morphological data available [13]. The uncertainty regarding the taxonomic status of *S. louisianae* warrants the need for further evidence to create a better understanding of the true boundaries of this taxa. Morphologically independent data like molecular and cytogenetics are of great value in this endeavor. In light of the absence of cytogenetic data for this species, the objective of this study was to present the first cytogenetic data for *S. louisianae*.

## Materials and Methods

**Figure 1:**
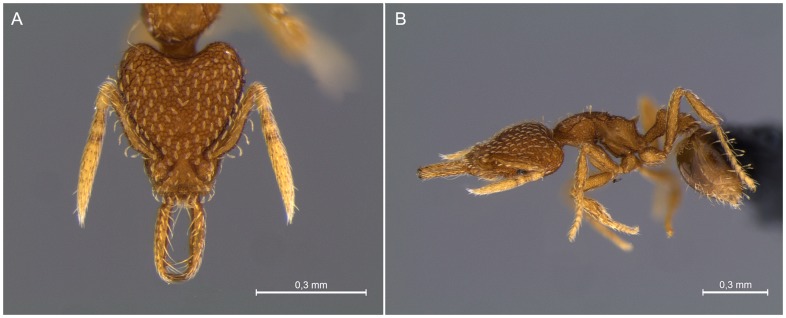
*Strumigenys louisianae* images: A) frontal view of the head, B) lateral view.

Cytogenetic studies were conducted on a *S. louisianae* colony collected in the ‘*Mata da Biologia*’ secondary forest patch located at the Universidade Federal de Viçosa campus, Viçosa, Minas Gerais, Brazil (20°45′23″S, 42°52′25″W) in July 2013. The national collecting permit was issued for Instituto Chico Mendes de Conservação da Biodiversidade - ICMBio to Luísa Antônia Campos Barros (SISBio: 32459-5).For this location specific permit was not required for the sampling and the species studied is neither an endangered nor protected species. The colony was maintained in a plastic container to obtain the larvae at an appropriate stage. One adult specimen was identified and photographed (Fig. 1) by Thiago Sanchez Ranzani da Silva and deposited in the Hymenoptera collection of the Museu de Zoologia, Universidade de São Paulo (MZUSP), Brazil.

The metaphases were obtained using the cerebral ganglion, according to Imai et al. [21]. More than 80 metaphases were analyzed from two individuals. Some metaphases were stained with 5% Giemsa, and 10 of these were measured for the classification of chromosome morphology as proposed by Levan et al. [22]. Characterization of the richness of the CG and AT base pairs along the chromosome was acquired using the fluorochromes Chromomycin A_3_ (CMA_3_) and 4′6- diamidino-2-phenylindole (DAPI), according to Schweizer [23].

## Results and Discussion

**Figure 2:**
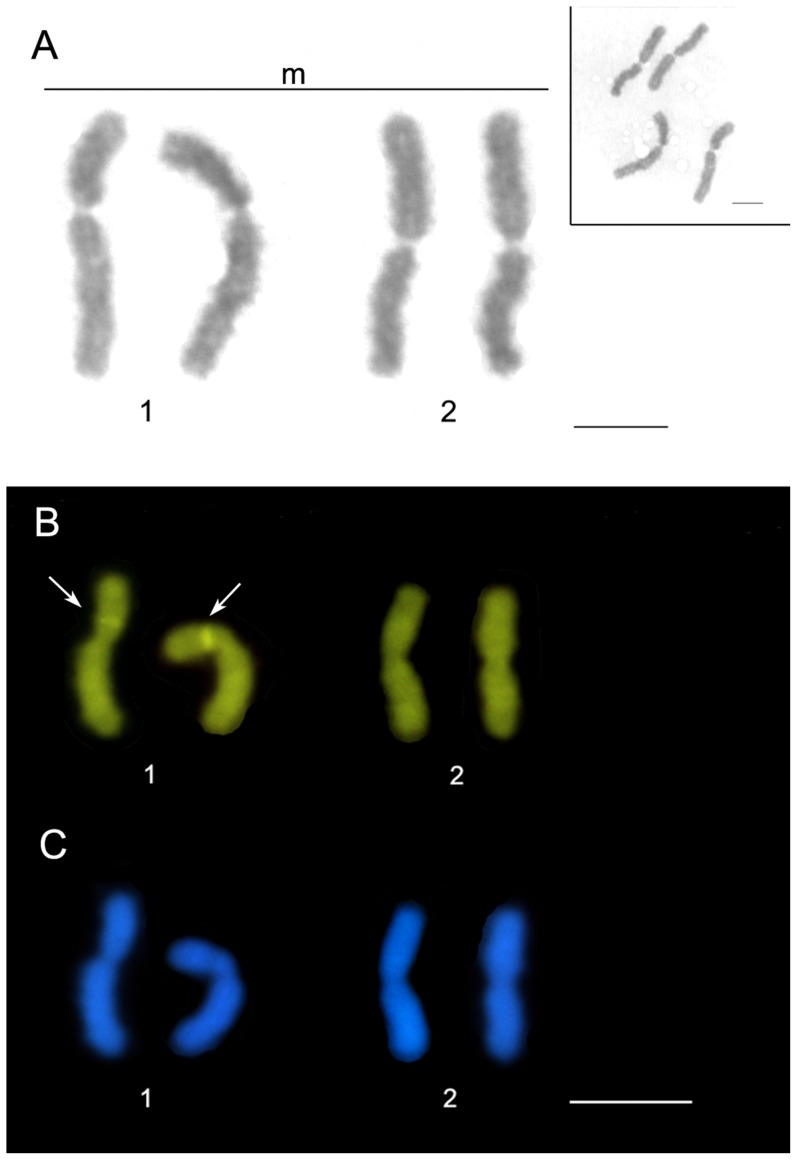
Metaphase and karyotype of the 2n = 4 chromosomes in *Strumigenys louisianae*; Giemsa staining (A) Karyotype with CMA_3_ (B) and DAPI staining (C). Arrows indicate the positive marks for CMA_3_. m =  metacentric. Bar: 5 µm.

*Strumigenys louisianae* presented 2n = 4 chromosomes (Fig. 2A), all metacentric and properly paired (mean of the arm ratio: first pair 1.61; second pair 1.04; as obtained from 10 metaphases). This species presents the lowest chromosome number among the Hymenoptera from the Neotropical region [4]. Data from this study also correspond to the lowest chromosome number ever recorded in the subfamily Myrmicinae. Previously, 2n = 8 chromosomes was considered the lowest number reported for this subfamily, which had been recorded for *Mycocepurus goeldii* [6] and *Mycocepurus* sp. [24]. Although a low number of cytogenetics studies were conducted on the Neotropical ant fauna, a great diversity rangeis observed,from the finding of 2n = 4 chromosomes in *S*. *louisianae* in this work to the highest number known for Hymenoptera, the 2n = 120 chromosomes found in *D. lucida* [8].

The occurrence of a species with a low chromosome number and being phylogenetically similar to others with higher numbers is not unique to the genus *Strumigenys*. A similar case is evident in the ant *Myrmecia croslandi* (Formicidae: Myrmicinae)in which 2n = 2 chromosomes were observed in the females, whereas in the males, which are haploid, the presence of n = 1 chromosome was found [25]. Although fission plays an important role in the evolution of the karyotype in Formicidae, some centric fusions occasionally occur as a mechanism for heterochromatin elimination, especially in those karyotypes presenting pseudo-acrocentric chromosomes [21]. A better supported interpretation, based on the cytogenetic and molecular data, for the emergence of the karyotype 2n = 2 chromosomes in *M. croslandi* suggests that this karyotype originated from the fusion of the chromosomes of the karyotype 2n = 4 occurring in individuals of the same species, where the intermediate karyotype 2n = 3 is known and possibly originated from the female gametes of *M. croslandi* with n = 2 chromosomes and the male with n = 1 chromosome or vice versa [25], [26]. *Pheidole nodus* presents chromosomal polymorphism in which its chromosomal number varies from n = 17 to n = 20, and an ancestral karyotype of n = 18; the other three karyotypes result probably either from centric fusion (n = 17) and fission (n = 19 and n = 20) [27]. Fusion type chromosomal rearrangements were also suggested recently in the evolution of the genus *Mycetophylax* [5]. Another example is the social parasite *Acromyrmex ameliae* that presents a distinct chromosome number of 2n = 36 rather than the 2n = 38 chromosomes, found in all other members of the genus *Acromyrmex* (2n = 38), indicating a centric fusion of two pairs of chromosomes (unpublished data). The same rearrangement has been suggested for vertebrate species, such as *Muntiacus muntjak* (Cervidae) during the formation of the karyotype 2n = 6 chromosomes in females and 2n = 7 in males from the karyotype 2n = 46 of *Muntiacus reevesi*. For the formation of the karyotype 2n = 6 it was suggested that at least 20 tandem fusions had to occur in the karyotype 2n = 46 [28]–[30]. With the cytogenetic data obtained to date for the genus *Strumigenys* (2n = 16 to 2n = 40, reviewed in [4]) it is believed that tandem fusions are possibly the ones responsible for the formation of the karyotype 2n = 4 observed in *S. louisianae*. It could have occurred as a mechanism of heterochromatin elimination, since the heterochromatic blocks were not evident in the chromosomes of this species on using the Giemsa staining protocol as proposed by [31].

The fluorochrome CMA_3_ showed the presence of a block rich in GC base pairs in the interstitial region of the short arm of the largest chromosome pair, where this is the first data recorded on banding in this genus (Fig. 2B). The CMA_3_ was used in some ant species and it revealed markings on a chromosome pair in *Dinoponera lucida* [8], *Azteca trigona* [32], and *Tapinoma nigerrimum* [33] corresponding to the Nucleolar Organizer Regions (NORs). This correlation was confirmed by the FISH and/or NOR banding technique. The correlation between the Nucleolar organizer regions (NORs) and GC-rich regions is a very common occurrence in Hymenoptera [34]; therefore, the banding with the CMA_3_ may contribute to the identification of the NORs, especially for the single NORs. These regions are considered conserved and found in specific locations for each species. As a result of this specificity, the description of the number and position of this region in the chromosomes can be reliably used in taxonomic and phylogenetic studies [35].

The fluorochrome DAPI nonspecifically marked the chromosomes of *S. louisianae;*however, the AT-rich regions were not observed (Fig. 2C). Some species among Hymenoptera present these markings, including some bees [34], [36] and the little fire ant *Wasmannia auropunctata* [35]. These markings are mainly present in the centromeric regions [37], although in *W. auropunctata* the DAPI rich regions were observed in the pericentromeric region in most chromosomes [35].

Morphology and Molecular Genetics are the most commonly used tools by systematists to reconstruct phylogeny. In this context, cytogenetics play an important role acting as another independent source of evidence that can strengthen ideas on the evolution of particular groups. Further cytogenetic data can bring evidence to the many synonyms that resulted in the current hyperdiverse genus *Strumigenys* [10]–[38] and shed light on the understanding of the evolution of the various mandible forms found in this genus. Moreover, data of this kind have the potential to test the contradictory concepts on the tribal rank of the Dacetini, Basicerotini and Phalacromyrmecini [10], [38]–[40], none of which are fully supported by the current molecular evidence [41], [42].
